# Brief Notes on Cholera in Louisville in 1850

**Published:** 1850-08

**Authors:** Theodore S. Bell

**Affiliations:** Louisville, Ky.


Art. II.—*Brief Notes on Cholera in Louisville in* 1850. By Theodore S. Bell, M. D., of Louisville, Ky.

The city of Louisville has been so much favored heretofore by exemption from epidemic disease, that it was deemed almost impossible that she could be visited by any serious outbreak. Twenty-eight years ago, when large ponds occupied the space that is now covered by populous squares, and a high summer temperature prevailed, she felt the scourging effects of a malarial season, and the spring and summer of 1850 are the first seasons within that period, in which a similar conjunction of heat and moisture has shown itself. We have not the space now to spare for recalling the former condition of things in this city to mind, but we shall do so hereafter. We announced more than a year ago that the extending cultivation of the land, in the pond region, adjacent to Louisville, had greatly improved the health of that region, and of that portion of the city which formerly suffered. And this judgement then expressed has been amply sustained by the severe trial to which the unusual season of 1850 has subjected it. With a season almost exactly similar to that of 1822, the pond settlement has been unusually healthy. More rain has fallen in this vicinity, in the spring and summer than in any year within our knowledge of Louisville, and we have known it eighteen summers. The temperature of the spring was remarkably low, and of the summer excessively high. The thermometer has ranged from 85° to 98°, and the air was very oppressive. But a large portion of Louisville was well prepared for even such a season as this. It was clean, well drained, and has escaped remarkably well. There were spots that were neither well drained, nor clean, and they suffered.

In the beginning of June, intermittent fever, dysentery, remittent fever, and cholera began to show themselves in low, filthy, or undrained localities. In one house, a family consisting of eight persons, living in the most wretched filth, on the banks of Beargrass, and all occupying the ground floor as lodging rooms, was attacked with cholera, and five of them died; the others were saved by being removed from the locality. A family, however, living next door, occupying the second story of the house as lodging apartments escaped without any sickness. Another family living in the same locality, removed to one no better. The house to which they removed was a new one, with two rooms, twelve by fourteen feet in size, and the plasterer had just finished his work on the rooms, and nine persons undertook to lodge in these two small rooms, with an atmosphere reeking with moisture from undried plastering. The removal took place on Saturday, on Sunday, the wife of one of the parties was taken sick and died with cholera. Her corpse was placed in one room, and the eight survivors undertook to sleep in the other. By Thursday but one of the nine was left alive, and his life was despaired of, but hearing of his situation, we had him removed to the hospital, and he recovered slowly. Sporadic cases of this kind continued to occur, and in the neighborhood of piles of decaying hemp offal, the cholera was especially malignant.

The writer of this, as a member of the Board of Health, made such inspections of the localities of cholera, as the arduous duties of an active practice would permit, and all possible aid was given to the city authorities in improving the sanitary condition of the upper end of the town. The citizens were urged to make personal inspections for themselves, because heavy rains, and a high temperature were continually varying the sanitary phases of various parts of the city, and a portion that might look well under a general inspection, might change materially under the influence of a rain.

On the 24th of July, we received an urgent note, requesting our immediate attendance upon a portion of the lower end of the city, where the pestilence had broken out with great violence. Professional engagements prevented immediate attention to this request, but immediately after dinner, in company with the Mayor we repaired to the scene of disaster, and examined it thoroughly. But some hours before this, orders had been given to cover the marshy strip of ground around an old pond, north-west of the locality of the pestilence, with sand, but this order was obeyed so inefficiently that the work all had to be done over again. The following is the topography of the locality referred to;

Market street between Tenth and Eleventh streets is the site of an old pond. Another pond, north-west of the square is still in existence, and a ropewalk is built on the eastern edge of this pond. An immense mass of refuse hemp had been thrown into the edge of the pond, and the evaporation of the water, had exposed a mass of this decaying vegetation to the action of the sun. A good deal of vegetable filth, in a moist condition, was on the east side of the ropewalk. The heavy rains of the spring and summer had so saturated the ground, between Tenth and Eleventh streets, that the cellars were generally full of water, and the filthy yards in that state of dampness that fitted them for the evolution of malaria, under the high temperature of the present summer. The ground was so saturated with water, that upon pushing the end of a walking stick down a few inches, water would come bubbling up. Pools of water stood under old frame houses that had no cellars, and all the walls showed the marks of extreme dampness. The sewer on Tenth street was choked, so that it could not carry off the water.

During the night of the 23d of July, cholera commenced its ravages in the small district we have described, and by Tuesday night at 11 o'clock there had been fifty cases, and thirty deaths. The remainder died after 11 o'clock, and during the next morning. From 11 o'clock on Wednesday, up to nightfall, there was not a new case, but immediately after sunset, there were fifteen new cases. On Wednesday and Thursday nearly all the survivors were removed out of the district, into healthy squares, and by the free use of sand and lime, the infected square was purified. A large number of citizens rushed to the scene of disaster, and labored diligently in the cause of humanity. They were assured that there was no danger in day-time, but that they must not expose themselves to the night air. Of the large number that worked in day time, from Tuesday up to Saturday afternoon, not one was attacked with disease. But a number of those who exposed themselves at night, died, thus giving conclusive proof of the malarial origin of cholera. A few of the survivors, who were scattered over the healthy parts of the town died at various periods, for more than a week, but no one of the families among whom they died, suffered any attack of cholera. We neglected to say in its proper place, that on the night the cholera broke out, the west wind was blowing over the decaying hemp in the edge of the pond, carrying the malaria directly upon the south side of the infected square, and the south side suffered much the greatest mortality.

But it is worthy of note, that the measures taken to destroy malaria, checked the cholera, so that it never extended beyond its first outbreak, and the endemic was brought to a close on the second day.

Two weeks after this, another outbreak took place towards the upper end of Jefferson street, in a square bounded by Preston and Jackson, Jefferson and Green. An old pond stands in the middle of the square, and its edges were covered with vegetable filth. The marshy places were in a horrible condition. On the north-west corner of the square is a number of miserable shanties, and back of them, extending the whole width of the lots on which they stand, was a green pool, containing masses of vegetable filth. The south-wind blew over this marsh, and the houses on both sides of Jefferson, and along on Jackson street, in the direct path of the wind suffered severely, while numbers of families on Green and Jackson, living much nigher the marshes escaped entirely. The wind blew the malaria from them over to Jefferson street. Under the floors of the shanties we have described, the water stood six feet deep, and nearly all the cellars in the square had from three and a half to five feet of water in them. About twenty deaths took place under this visitation, but they were scattered over several squares. A resort to the measures that had been successful in the lower end of town, was made, and they succeeded in the same prompt and efficient manner. The malarial origin of cholera has been as clearly demonstrated in this city, this summer, as if it were written, as it was, by sun-beams.

The square of which we have spoken suffered from cholera in 1833, and we described it at that time. Other squares which suffered then have been so improved, that they no longer make malaria, and they have escaped this season as well as other healthy places have. In fact there is not a dry, airy, clean square in the city that has not been as free from cholera, as it has from Oriental plague.

These are facts of importance, and should impress themselves everywhere upon the public mind. Those places in Louisville, which bore the brunt of the cholera in 1833, and which have been improved so as to be dry, clean, and airy, did not have a case of cholera in them in 1849, or 1850, except a single case in one of these improved squares. But those places in this city, which were scourged in 1833, and which remain now, in the state they were in then, have been scourged again in 1850. Apply these plain and practical remarks to districts or states, and their sanitary bearing is two obvious to need anything from us.

We hope to be able to give our readers, in our next number, a more detailed view of these matters.

